# Mo/Si multilayer-coated amplitude-division beam splitters for XUV radiation sources

**DOI:** 10.1107/S0909049512049990

**Published:** 2013-01-23

**Authors:** Ryszard Sobierajski, Rolf Antonie Loch, Robbert W. E. van de Kruijs, Eric Louis, Gisela von Blanckenhagen, Eric M. Gullikson, Frank Siewert, Andrzej Wawro, Fred Bijkerk

**Affiliations:** a FOM-Institute DIFFER, Edisonbaan 14, 3439 MN Nieuwegein, The Netherlands; b Institute of Physics, Polish Academy of Sciences, Aleja Lotników 32/46, 02-668 Warsaw, Poland; c Carl Zeiss SMT GmbH, Rudolf-Eber-Strasse 2, 73447 Oberkochen, Germany; d Center for X-ray Optics, Lawrence Berkeley National Laboratory, Berkeley, CA 94720, USA; e Helmholtz Zentrum Berlin/BESSY-II, Albert-Einstein-Strasse 15, 12489 Berlin, Germany; f Mesa+ Institute for Nanotechnology at the University of Twente, PO Box 217, 7500 AE Enschede, The Netherlands

**Keywords:** Mo/Si, multilayer, XUV, beam splitter, free-electron laser, amplitude division

## Abstract

Amplitude-division beam splitters for XUV radiation sources have been developed and extensively characterized. Mo/Si multilayer coatings were deposited on 50 nm-thick SiN membranes. By changing the multilayer structure (periodicity, number of bilayers, *etc.*) the intensity of the reflected and transmitted beams were optimized for selected incident radiation parameters (wavelength, incident angle). The developed optical elements were characterized by means of XUV reflectometry and transmission measurements, atomic force microscopy and optical interferometry. Special attention was paid to the spatial homogeneity of the optical response and reflected beam wavefront distortions. Here the results of the characterization are presented and improvements required for advanced applications at XUV free-electron lasers are identified. A flatness as low as 4 nm r.m.s. on 3 × 3 mm beam splitters and 22 nm r.m.s. on 10 × 10 mm beam splitters has been obtained. The high-spatial-frequency surface roughness was about 0.7–1 nm r.m.s. The middle-spatial-frequency roughness was in the range 0.2–0.8 nm r.m.s. The reflection and transmission of the beam splitters were found to be very homogeneous, with a deviation of less than 2% across the full optical element.

## Abstract 2

Short-wavelength XUV beam splitters consisting of multilayer reflective and transmissive coatings on 3 × 3 mm and 10 × 10 mm SiN membranes have been developed and fully characterized.

## 1. Introduction

Multilayer (ML) coated optics (Louis *et al.*, 2011 ▶) are used to control extreme ultraviolet (XUV, also called EUV) and soft X-ray (SXR) radiation in many fields of science and technology, such as advanced photolithography, X-ray fluorescence analysis and space research. A rapidly developing field is the application in experiments at short-wavelength free-electron lasers (FELs), the new generation of very intense XUV-based radiation sources. A particularly appealing but challenging application is a ML-based XUV beam splitter (BS), the fabrication and characterization of which will be described in this paper.

Such beam splitters based on multilayer-coated membranes (ML-BSs) are required for, for example, pump–probe experiments, parallel user operation at FELs, autocorrelation measurements to determine the time structure of FEL pulses (Mitzner *et al.*, 2008 ▶, 2009 ▶), online wavefront diagnostics (Zeitoun *et al.*, 2004 ▶), ellipsometry (Kortright *et al.*, 1992 ▶; Kimura *et al.*, 2005 ▶), polarimetry (Schäfers *et al.*, 1999 ▶), interferometry (Da Silva *et al.*, 1995*a* ▶; Smith *et al.*, 2003 ▶), transmission filters (Volodin *et al.*, 2010 ▶), Fourier transform spectroscopy (de Oliveira *et al.*, 2011 ▶) and as output couplers for laser cavities (Stearns *et al.*, 1986 ▶; Ceglio, 1989 ▶).

Beam-splitting techniques for the XUV exist in the form of wavefront splitting [*e.g.* Fresnel’s double mirror and Lloyd’s mirror (Rus *et al.*, 2001 ▶; Rocca *et al.*, 1999 ▶), edge mirror splitting (Mitzner *et al.*, 2008 ▶, 2009 ▶; Schlotter *et al.*, 2010 ▶; Roling *et al.*, 2011 ▶, 2012 ▶), half-splitted mirror (Moshammer *et al.*, 2011 ▶)] and amplitude splitting [*e.g.* diffraction gratings (Goulielmakis *et al.*, 2002 ▶) or semitransparent membranes]. The main drawbacks of wavefront-splitting techniques are the sensitivity to pointing instabilities and the disturbance of wavefronts by diffraction at the sharp edges. Amplitude splitting *via* semitransparent membranes overcomes these problems and, in addition, allows for an adjustable reflection-to-transmission ratio when using ML coatings. Moreover, such components are necessary for some optical schemes like Michelson interferometers. However, these ML-BSs are very fragile and more difficult to make, especially with respect to the flatness necessary to preserve the reflected wavefront of the BS.

Different research groups have attempted to fabricate ML-based XUV semitransparent BSs, either free-standing or supported on a thin membrane (*e.g.* silicon nitride). However, in their publications there is limited characterization and description of the properties of the ML-BSs.

In this paper we present the results of a broad characterization of amplitude-division BSs with supporting membranes (both in transmission and reflection) for the new generation of XUV/SXR light sources. We also discuss important aspects and identify problems for further development for more advanced applications such as Fabry–Perot-like interferometry that uses two closely spaced BSs. Furthermore, we present a literature overview of fabricated ML-BSs up to now.

## 2. Current state of ML-BS technology

Different approaches of ML-BSs have been applied in the past. Table 1 ▶ shows an overview of different types of BS, properties on roughness and flatness, and results on reflectance and transmittance. It should be noted that there is only one publication (Haga *et al.*, 1998 ▶) that fully describes the fabrication process and also fully characterizes the produced (free-standing) BSs.

There are various methods to prepare ML-BSs, which can be categorized into two groups: free-standing MLs or MLs supported on a membrane. The preparation always includes a substrate, typically highly polished silicon, which is coated with silicon nitride, silicon carbide or a photoresist. The ML structure is then deposited onto the coated Si substrate. The coated substrate in some cases has been etched from the back prior to the ML deposition leaving a window with a thin SiN or SiC membrane (30–890 nm) supporting a ML coating (Schäfers *et al.*, 1999 ▶; Khan Malek *et al.*, 1989 ▶; Di Fonzo *et al.*, 1994 ▶; Nguyen,1994 ▶; Da Silva *et al.*, 1995*b* ▶; Tinone *et al.*, 1996 ▶; Wang *et al.*, 2003 ▶, 2007 ▶). Sometimes this free membrane is coated on both sides (Zeitoun *et al.*, 2004 ▶; Hubert *et al.*, 2001 ▶; Smith *et al.*, 2003 ▶). In other cases the Si substrate coated with SiN or SiC is back-etched after the ML deposition, leaving the ML on the SiN or SiC membrane (Stearns *et al.*, 1986 ▶; Ceglio, 1989 ▶). In order to make a free-standing ML, the SiN or SiC membrane is also etched away, *e.g.* with dry-reactive ion etching (Haga *et al.*, 1998 ▶, 2000 ▶; Takenaka *et al.*, 2005 ▶; Andreev *et al.*, 2005 ▶; MacDonald *et al.*, 2008 ▶, 2009 ▶). Another method to make free-standing ML-BSs is by using the photoresist or another sublayer that is dissolved and the ML is subsequently lifted off and remounted onto a frame (Kortright *et al.*, 1992 ▶; Kimura *et al.*, 2005 ▶; Volodin *et al.*, 2010 ▶; Nomura *et al.*, 1992 ▶).

The lift-off and remount procedure, however, causes a large waviness of the BS. Thus this type of BS has corresponding limitations for practical use in most of the experimental set-ups. The other mentioned techniques result in much flatter ML-BSs, with peak-to-valley (P–V) values in the range of nanometers to micrometers. The advantage of having a ML on a membrane is that is gives a larger stiffness to the fragile ML. Moreover, such membranes are commercially available which eliminates the etching steps and simplifies the BS technology. However, for XUV and SXR radiation these SiN and SiC membranes strongly absorb, and hence induce large losses to the pulse energy. This limits the usable thicknesses of the membranes. In addition, these membranes have a higher surface roughness then polished Si wafers, which affects the growth of the ML, typically resulting in larger interface diffusion and roughness. In the case of free-standing MLs there is no problem with the increased absorption in the support, but the use of a rough SiN or SiC coating as substrate still decreases their optical performance. Furthermore, the removal of the support after the ML deposition leads to damage of the ML itself. Overcoming that issue has led to the use of Ru layers on both sides of the ML, but this again induces a loss in photon flux due to high absorption (Haga *et al.*, 1998 ▶).

Most ML-BSs are used as tools for other experiments and are only briefly described in the publications. Only Haga *et al.* (1998 ▶) have extensively characterized the free-standing ML-BS window. They also fabricated the flattest BS down to 1 nm r.m.s. on a 7 × 7 mm surface area with low ML stress, and with a high reflectivity and transmission of both 27%. However, they did not include the complete frame in their characterization, which is important for applications with closely spaced BSs.

We decided to use thin (50 nm) SiN membranes as substrates for ML-BSs to avoid etching steps and characterize these more extensively and identify possible limitations and optimization steps.

## 3. Experimental

The substrates for ML reflecting coatings were provided by Silson Ltd. They consist of a silicon-rich nitride (SiRN) film (non-stochiometric, optimized for low stress) supported by a monocrystalline silicon frame. Studied membranes were 50 nm thick [according to the manufacturer, across a single membrane, thickness uniformity was better than 1%, with ∼200–300 MPa tensile stress in a 100 nm-thick film (private communication)] and had a square shape with 3 × 3 mm or 10 × 10 mm dimensions. The window was formed by means of wet anisotropic etching in a 381 µm-thick silicon wafer. The delivered frames had a square shape of 5 × 5 mm and 17.5 × 17.5 mm dimensions, for smaller and larger windows, respectively.

Mo/Si MLs were used as a reflecting coating (Louis *et al.*, 2011 ▶). The ML structure (period, materials’ thickness ratio Γ, number of bilayers) was optimized for several applications using 13.5 nm radiation by means of optical response simulations with *IMD* software (Windt, 1998 ▶). The 13.5 nm wavelength was chosen as it is one of the most common available wavelengths at XUV sources [*e.g.* plasma-based lasers (Barkusky *et al.*, 2010 ▶), high-harmonic generation (Chen *et al.*, 2010 ▶; Loch *et al.*, 2011 ▶), free-electron laser at Hamburg, FLASH (Ackermann *et al.*, 2007 ▶)] and there is a large available expertise on ML coatings coming from lithography research. The coatings were deposited by electron-beam evaporation in an UHV background of ∼1 × 10^−8^ mbar, with post-deposition smoothing of the Si layers using low-energy ion treatment. In parallel to the membranes, optically polished (roughness below 0.2 nm r.m.s.) monocrystalline silicon wafers were coated. Two of them were pre-characterized by means of optical interferometry and were later used for measurements of the stress induced by the coatings by evaluating the changes in the surface shape before and after ML deposition (so-called ‘stress wafers’). At least one additional silicon wafer was used as a reference sample for XUV and X-ray reflectometry.

**Figure 1:**
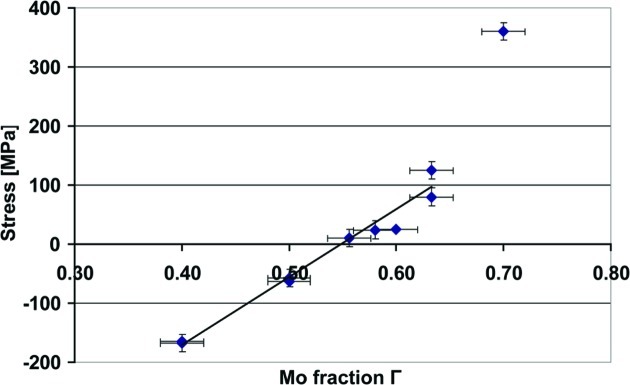
Stress induced by 30 bilayer Mo/Si ML coatings on a superpolished Si wafer using electron-beam deposition and varying the Mo fraction. Negative stress values correspond to compressive stress, positive values to tensile stress. The optimum Γ for ML-BSs on 50 nm SiRN is ∼0.63.

**Figure 2:**
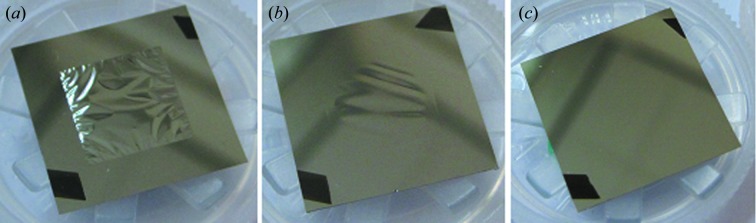
ML-BSs having (*a*) compressive stress in the ML as measured on Si stress wafers (Γ = 0.4), (*b*) zero-stress ML, and (*c*) ∼100 MPa tensile stress in the ML.

The stress in Mo/Si MLs was tuned by varying the fraction of Mo (Γ) in the ML structure (Zoethout *et al.*, 2003 ▶). We optimized the stress by making various coatings consisting of 30 bilayers with different Γ and inspecting the flatness of the BS visually. In Fig. 1 ▶ the stress induced by the ML coatings on a bulk silicon substrate is shown with respect to the Γ parameter. Fig. 2 ▶ shows photographs of a ML-BS for three cases with different internal stress. The BS shows wrinkles for compressive stress (Fig. 2*a* ▶) and also around zero stress (Fig. 2*b* ▶). Some BSs with a zero-stress ML appear flat immediately after the deposition but later form wrinkles. For large tensile stress, at Γ = 0.7, all of the membranes break very early during the coating process. Around an aimed Γ of 0.633 we obtain the flattest BSs, illustrated in Fig. 2(*c*) ▶. By using this optimized Γ, typically three to four out of the four coated membranes survive after the deposition process. From Fig. 1 ▶ it can be seen that for a fixed Γ of 0.63 there is some fluctuation in stress around 100 MPa. Using unchanging coating parameters and aiming for Γ = 0.633, the final Γ as determined by X-ray reflectometry (critical angle of the Cu *K*α line) varies between 0.63 and 0.66, which slightly influences the stress. It should be noted that the stress wafers coated with a different number of bilayers show different stress values (normalized to the film thickness). We observed that for a Γ of 0.633 the stress value reduces with increasing number of bilayers. Also, for five and 12 bilayer BSs there are no wrinkles observed for Γ = 0.5. Furthermore, the values of stress in the ML film on the thin SiN membrane will be different, because Stoney’s law (Stoney, 1909 ▶) used to calculate the film stress on the silicon substrate only holds when the substrate thickness is much larger than the film thickness.

The stress-optimized BS samples were further characterized using different methods, described below. The performance of the BS, *i.e.* the reflectance, transmittance and wavefront propagation, are affected by the ML structure, the roughness and shape of the BS.

High-spatial-frequency roughness (microroughness) of the substrate affects the growth of the ML. In particular, for Mo/Si a rough substrate results in the growth of rougher interfaces with a larger interdiffusion at the layer interfaces, which lowers the reflectivity of the ML. This frequency range can typically be measured by means of atomic force microscopy (AFM). We characterized the surface roughness on uncoated and coated membranes. Mid-spatial-frequency roughness (waviness) affects the radiation scattering and interferes with the wavefront. This surface roughness of uncoated and coated membranes was characterized using white-light interferometry with a Mirau and a Michelson optics set-up. The low spatial frequencies (profile, figure or shape) of the surface are responsible for the main wavefront deformations, specified through Zernike terms. The BS shape was studied by means of a 4" Fizeau-type interferometer (ZYGO-GPI) (Hariharan, 1985 ▶). It is worth noting here that we qualify a BS to be of good quality (diffraction-limited) when the wavefront errors are lower than λ/14 r.m.s., λ being the radiation wavelength, according to the Marechal criterion (Zeitoun *et al.*, 2004 ▶).

The optical response of the ML-coated membranes was measured at the Center for X-ray Optics, Berkeley, USA, using 90% s-polarized XUV radiation with a beam size of ∼0.3 mm × 0.05 mm from the Advance Light Source. The reflectivity and transmission were measured for different incident angles around the resonance angle at 13.5 nm wavelength. For the measured resonance angle additional wavelength scans of the reflectivity and transmission were made at a few different locations. At 13.5 nm wavelength and the resonance angle, two-dimensional reflectivity and transmission maps were measured.

## 4. Results

The structure of the ML-BSs was optimized for the following applications (see Table 2 ▶) and were characterized by the techniques described above. A first application was the pump–probe set-up foreseen for microfocusing experiments at the FLASH facility. The BS is used to couple out part of the incident beam and part of the reflected beam after interaction with the studied material to photodiodes to measure the pulse intensities. To maximize the signal on the probe photodiode one needs to maximize the product of the BS reflectivity and transmission. This was the main criterion in the design, apart from the 13.5 nm wavelength of the FLASH radiation and ∼15° incident angle together with p-polarization, forced by the set-up geometry. The second design was optimized for a BS in a Mach–Zehnder interferometer. In this case the ratio of the reflectivity over the transmission should be close to unity and the incident angle close to 45°. Since at such an angle and wavelength (13.5 nm) the p-polarized light is reflected very weakly, the BS can be used for s-polarized light only.

Another application is a Fabry–Perot-like interferometer (IFO) meant for autocorrelation measurements of the time structure of FEL pulses in single-shot mode, or for Fourier transform spectroscopy. The IFO contains two very closely spaced BSs, down to submicrometer distances apart. Therefore, very flat BSs are required including the edge of the supporting frame. For first experiments, we designed the IFO to be used around 12.5° for s-polarization. Here, the criterion for reflection and transmission is that the intensity of the reflected beam off the first BS should be equal to the reflected beam off the second BS multiplied by the double transmission through the first BS. This resulted in using five and 12 bilayers on the 50 nm-thick SiN membranes with a period thickness of 7.3 nm. In yet another considered design we maximized the number of bilayers (30) of the ML coating to obtain maximum intensity of the reflected beam.

The designed coating parameters were obtained by changing the bilayer period *d*, the materials’ thickness ratio Γ and the number of bilayers *N*. These parameters were studied in advance by means of optical response simulations using *IMD* software (Windt, 1998 ▶). The results of the characterization were then used as a feedback for the design process.

**Figure 3:**
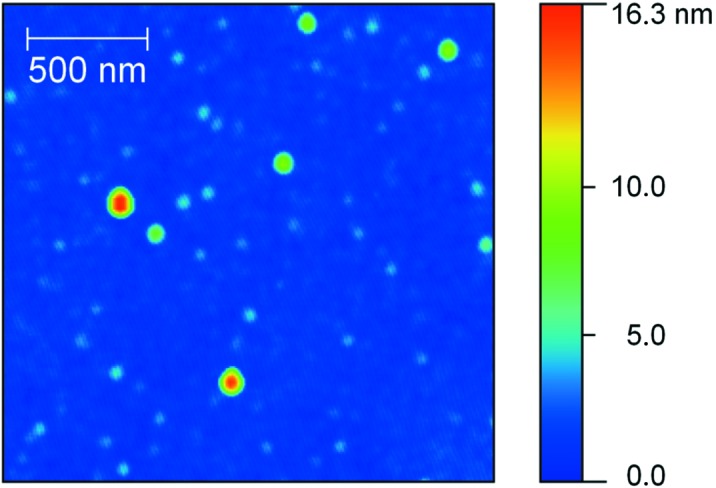
Example of an AFM topography map (2 × 2 µm) on a 3 × 3 mm coated membrane. The r.m.s. of the surface roughness is equal to 1.0 nm. The vertical scale is marked with a colorbar.

The surface roughness at high spatial frequencies (4 × 10^5^–2×10^8^ m^−1^, *i.e.* 5 nm resolution over 2.5 × 2.5 µm image size) is an important parameter for the BS design and was measured by means of AFM. Topography maps were made for both uncoated and coated membranes surfaces. The topography of a ML-coated membrane is shown in Fig. 3 ▶. No significant difference was noticeable between the spots located at the frame and at the window positions. The obtained surface roughness was equal to 1.4 nm and 0.7–1 nm for uncoated and coated membranes, respectively. It is much higher than for the standard optically polished silicon substrate (where it is less than 0.2 nm) and influences the optical response of the studied samples. The decrease of the surface roughness for coated membranes as compared with the uncoated ones can be attributed to the smoothing of the silicon layers by ion polishing during the deposition process.

**Figure 4:**
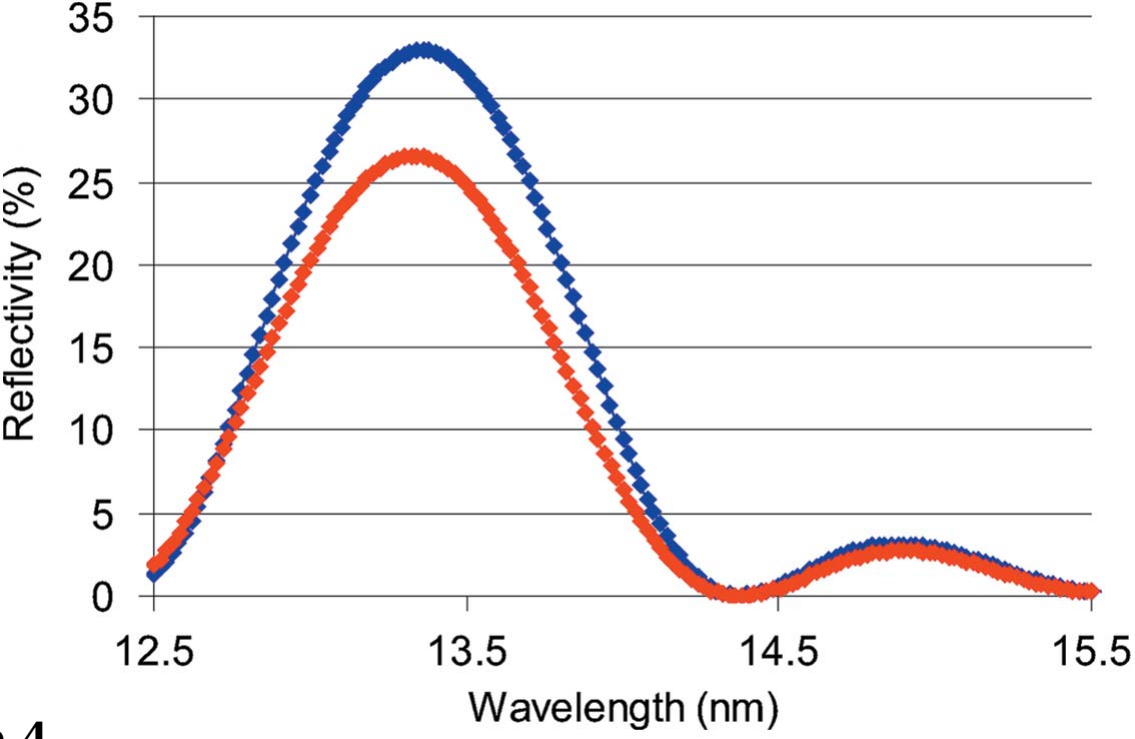
Measured reflectivity curves for ML coatings with *d* = 7 nm, *N* = 12 and Γ = 0.5 on a superpolished Si wafer (blue curve) and on a 50 nm SiN membrane (red curve) with a higher surface roughness.

The difference in optical response resulting from the roughness is illustrated in Fig. 4 ▶, where the blue curve shows the ML coated on a superpolished Si reference wafer and the red curve shows the reflectivity of the same ML coating on a SiN membrane. The optical response of the reference sample (coating on the polished Si wafer) fits very well to the ML simulations made by the *IMD* software. In the model of the ML structure an interdiffusion layer’s thickness within a bilayer equal to 1.6 nm was used, with layer roughness σ equal to 0.2 nm, and the optical constants were assumed to be the same as for bulk materials. In contrast to the ML on polished Si, the optical response of the ML-coated membrane is significantly different, with a relative drop in peak reflectivity of 20% (6% absolute drop) and a small shift in the peak wavelength. This can be explained by the enhanced roughness of the membrane and consequently of the deposited layers with larger interdiffusion at the interfaces.

**Figure 5:**
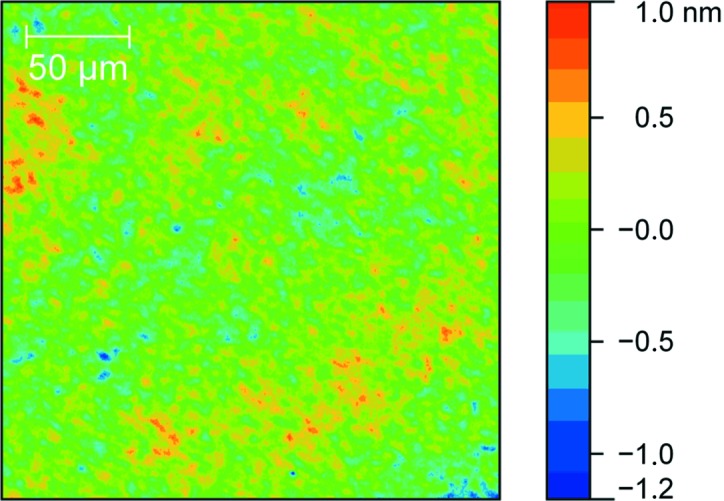
Example of an optical interferometry surface topography map of a 235 × 235 µm spot (measured with a magnification of 20×) on the 3 × 3 mm coated membrane. The r.m.s. of the surface roughness is equal to 0.2 nm. The vertical scale is marked with a colorbar.

In the next step of the BS characterization, we used white-light interferometry with Mirau optics to measure the mid-spatial-frequency roughness of the uncoated and coated membranes. We used two different magnification factors in the Mirau microscope, namely 20× (field of view 235.2 × 235.2 µm, 480 × 480 pixels) and 50× (field of view 94 × 94 µm), which probe the spatial frequency ranges of 4 × 10^3^ to 2×10^6^ m^−1^ and 1 × 10^4^ to 5 × 10^6^ m^−1^, respectively. An example of a topography map is shown in Fig. 5 ▶. The surface roughness of 3 × 3 mm and 10 × 10 mm membranes coated with 30 bilayers was measured to be in the range 0.2–0.8 nm r.m.s. The variation in roughness values for the different coated membranes is caused by different initial roughness of the SiN membranes and might also be due to small variations of the ML deposition parameters. The results for one deposition run (coatings of a different number of bilayers on subsequent coating days) on 10 × 10 mm membranes are given in Table 3 ▶. The uncoated membranes appear to have a slightly smaller surface roughness than the coated membranes. Also, for the higher-spatial-frequencies range, one could observe a small increase in roughness for an increasing number of bilayers. Nevertheless, in all cases the values for the surface roughness meet the Marechal criterion and are well below the λ/14 r.m.s. value. This means that radiation scattering does not perturb the optical response of the BS.

We characterized the optical performance of a BS at several positions, and also constructed a two-dimensional map of the reflectivity and transmission. We discuss the results on an example of a sample designed for a selected application, *i.e.* the 1:1 BS working at 13.5 nm wavelength under 47.5° incident angle. The conclusions, however, are valid for all samples studied.

**Figure 6:**
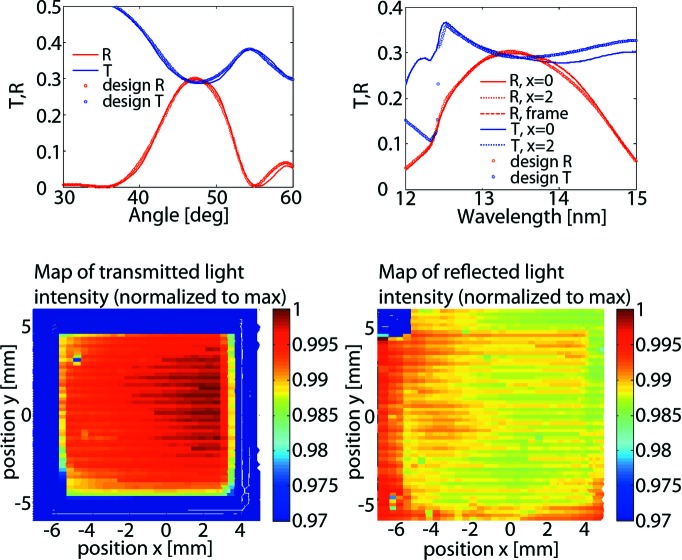
Transmission and reflection properties of a BS with *N* = 5, Γ = 0.48 and *d* = 10.6 nm. Across the whole BS window there is less than 2% deviation relative to the signal maximum.

Fig. 6 ▶ shows the reflectivity *R* and transmission *T* as a function of the incidence angle at 13.5 nm wavelength (top left) and as a function of the wavelength at 47.5° angle (top right), for both the measured values and the design values. It can be seen that the measured *R* and *T* correspond well with the designed values, which took into account the larger interfaces owing to the rough SiN membrane surface. In the lower images of Fig. 6 ▶ the reconstructed two-dimensional maps are shown of the transmission (left) and the reflectivity (right) from the 10 × 10 mm window and part of the frame. Inside the window the deviation is less than 2% (relative to the signal maximum). Thus it can be assumed that the reflection and transmission are very uniform across the full BS area.

**Figure 7:**
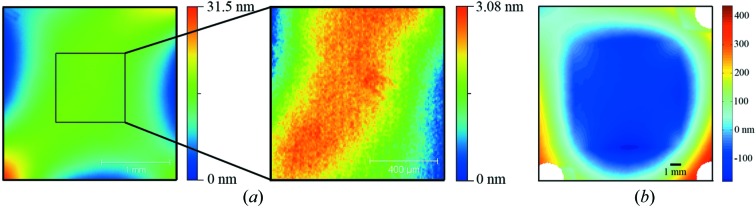
(*a*) Two-dimensional map of the surface shape of a 3 × 3 mm BS with 12 bilayers and the center zoom (1 × 1 mm), having 4.2 nm r.m.s. and 0.65 nm r.m.s., respectively. Only part of the window meets the Marechal criterion. (*b*) Profile of the flattest 10 × 10 mm BS with five bilayers characterized with the full frame.

In order to characterize the influence of the BS on the (reflected) wavefront, we also measured the shape of the BS (low spatial frequencies) using a ZYGO Fizeau-type interferometer; the quality of the reference flat used was λ/100 (at λ = 633 nm). A typical example of a 3 × 3 mm coated membrane is shown in Fig. 7(*a*) ▶, with the whole BS window on the left and a zoom of the center (1 × 1 mm) on the right. In this example the r.m.s. profile of the sample surface is equal to 4.2 nm on the full window and 0.65 nm r.m.s. on the 1 × 1 mm center area. For all BSs (3 × 3 mm and 10 × 10 mm) only a small area meets the Marechal criterion. The whole window is too largely curved, especially in case of the 10 × 10 mm BS. These BSs show a large curvature owing to the coating with P–V values of 200 nm to 1 µm. Furthermore, these values are even larger when the full frame is considered as well, up to 3 µm. Fig. 7(*b*) ▶ shows the shape of the flattest large BS having five bilayers. Here, the full frame has a P–V of 670 nm (112 nm r.m.s.) and the 10 × 10 mm window has a P–V of 200 nm (22 nm r.m.s.).

**Figure 8:**
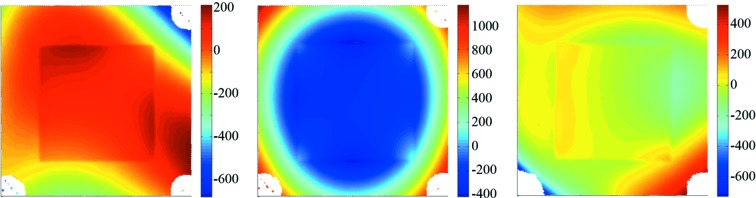
Two-dimensional map of the surface shape of three different uncoated SiN membranes. The vertical scale (in nm) is marked with a colorbar. The P–V and r.m.s. values are given in Table 4 ▶.

The problem of the non-flatness of the BS can be partly attributed to the SiN membranes. The membranes appear to be flat but in fact have a large irregularity and also deviate in shape from sample to sample, as is illustrated in Fig. 8 ▶ and Table 4 ▶.

**Figure 9:**
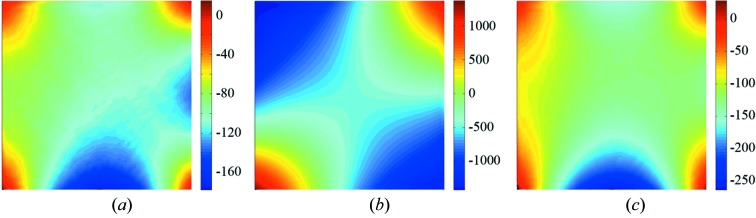
Effect of mounting on the shape of a 10 × 10 mm BS. (*a*) The BS when loosely supported (reference), (*b*) when fixed using two clamps, and (*c*) when fixed using minimal stress in a specially designed holder. The vertical scale (in nm) is marked with a colorbar. The r.m.s. and P–V values of the shapes are given in Table 5 ▶.

Another problem arises when the BS is mounted on a holder in experimental set-ups. Because the frame is very thin (of the order of hundreds of micrometers), small forces can lead to large deformations. This is illustrated in Fig. 9 ▶. The shape (10 × 10 mm) of the five bilayer coated membrane, as in Fig. 7(*b*) ▶, is shown when loosely supported, *i.e.* no pressure on the frame (*a*), when fixed using standard clamps on two opposite corners (*b*), and when fixated by means of a specially designed holder for use in our Fabry–Perot-like interferometer where the pressing forces are minimized (*c*). This is done by carefully fine-tuning the pressing force on three corners individually using adjustable metal springs. In Table 5 ▶ the values for the shape in r.m.s. and P–V are given for the full BS including frame, the window only, and for zooms of 5 and 1 mm at the center.

The clamps deform the BS shape dramatically and increase the values by an order of magnitude. The special holder, on the other hand, preserves the shape very well, and even slightly compensates locally (1 × 1 mm) the flatness of the BS.

**Figure 10:**
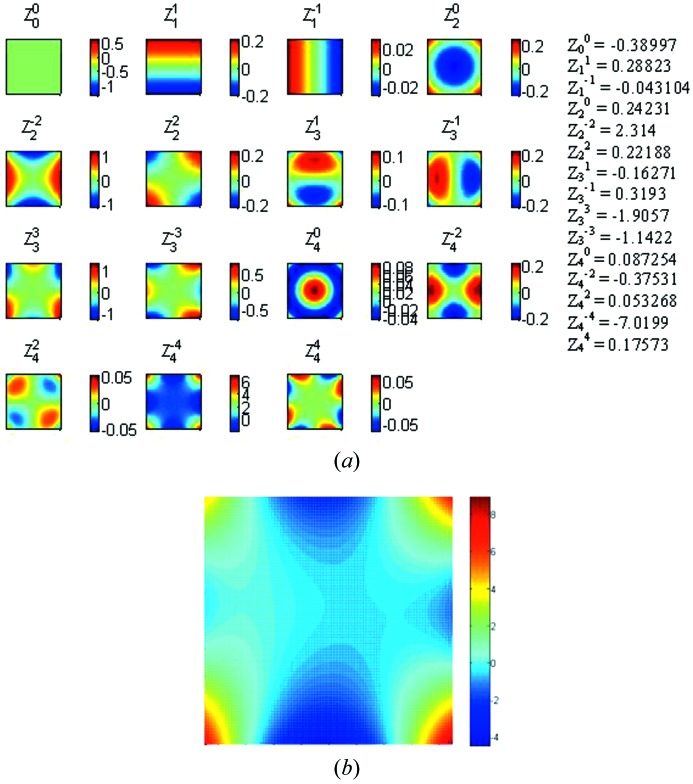
Zernike polynomials of the BS in Fig. 9(*a*) ▶, and reconstructed image using 15 polynomials.

A BS is a suitable tool to measure online a photon beam’s wavefront. In order to deduce the wavefront of a beam, one can compensate the measured wavefront after reflection off the BS with the measured profile of the BS itself, which can be specified through Zernike terms using 15 polynomials as illustrated in Fig. 10 ▶. Here the coefficients are given for the example of Fig. 9(*a*) ▶. The image in Fig. 10(*b*) ▶ is the reconstructed image using these 15 polynomials.

## 5. Conclusions and discussion

We have developed short-wavelength XUV beam splitters consisting of multilayer reflective and transmissive coatings on 3 × 3 mm and 10 × 10 mm SiN membranes and have fully characterized them, including the supporting frame as well as the mechanics to clamp the BS. We identified the useful properties and limitations, and optimized the stress inside the ML by tuning Γ to obtain a smooth BS. The optical performance is influenced by the rough surface of the SiN membranes, an effect which was included in the BS design. The reflection and transmission are uniform across the BS window with less than 2% deviation. The reflection and transmission can be varied for different applications by changing the ML structure. The mid-spatial-frequency roughness meets the Marechal criterion and is therefore not a limiting factor on the performance. The shape (or low-frequency roughness), however, is largely curved with high-order Zernike terms, which limits the use in applications where wavefront preservation is required. Locally, on a selected area of the order of 1 mm^2^, the BSs are sufficiently flat to meet the Marechal criterion. We fabricated BSs with flatnesses as low as 4 nm r.m.s. (32 nm P–V) on a 3 × 3 mm window, and 22 nm r.m.s. (200 nm P–V) on a 10 × 10 mm window, which are similar values to those reported in the literature on ML-BSs on SiN membranes. These values can to a large extent be attributed to the non-flatness of the SiN membranes. The shape of the SiN membranes was also found to differ for each sample, necessitating pre-characterization of the uncoated membranes.

There are several important aspects for further optimization of the membrane-supported ML-BSs. First of all, using thicker Si frames to support the SiN membranes could result in flatter membranes and hence in flatter ML-BSs, and should also lead to smaller deformation when fixed in a holder. In addition, round windows could result in more uniform stresses in the SiN membranes and therefore in flatter samples. Furthermore, for use in FEL applications, studies should be performed on the stability in time of the shape and optical performance and on the heat loads.

Nevertheless, our ML-BSs have met the requirements for a wide range of applications where wavefront preservation is insignificant.

## Figures and Tables

**Table 1:** Literature overview of fabricated ML-BSs

ML	Support	Size	Surface roughness r.m.s.	Flatness	Stress (MPa)	*R* (%)	*T* (%)	Θ (°)	λ (nm)	*d* (nm)	Γ	*N*	Ref†
Mo/Si	30 nm Si_3_N_4_					18	4	0.5	20.8	11.4	0.4	7	*a*
Mo/Si	300 nm SiN	10–20 mm^2^				13.4		0.5	12.7	6.5	0.4	13	*b*
	44 nm SiN									11.5	0.51	7	
	30 nm SiN					10	45	0.5	13.1	7.1		11	
Mo/C	300 nm SiC	10 × 10 mm			−800	6	0.45	78.5	1.33	3.2	0.34	35	*c*
Mo/Si	Free-standing	8 mm diam		‘Reasonable’			10	42	12.8	9.02	0.34	40.5	*d*
							14	46	12.8	9.02	0.34	40.5	
Mo/Si	Free-standing		0.65 nm	‘Not perfectly flat’			20	49	12.8	8.75	0.33	20	*e*
Cr/C	500 nm Si	3 × 5 mm	0.65 nm				22	46.59	4.68	3.25	0.33	50	*f*
Mo/Si	100 nm SiN	12 × 12 mm		500–1000 nm P–V		20	15		15.5	7.98		8–12	*g*, *h*
Mo/Si	150 nm SiN	2.5 × 2.5 mm				15	10	45	13.6			6	*i*
Mo/Si	200 nm SiN	10 × 10 mm			50					9.51	0.35	20	*j*
Mo/Si	Free-standing	10 × 10 mm	Top 1.03–1.24 nm	5 nm r.m.s. (10 × 10 mm)	50	27	27	45	13.4	10	0.36	5–10	*k*, *l*
			Interface 1.55–1.47	1.1 nm r.m.s. (7 × 7 mm)									
			Bottom rougher										
Mo/Si	50 nm Si_3_N_4_	2 × 2 mm	1.6 nm	Waviness						9.4	0.33	50	*m*
Cr/C	120 nm Si_3_N_4_	3 × 5 mm								3.2	0.33	10	
Cr/Sc	120 nm Si_3_N_4_	3 × 5 mm				4.5				1.57	0.66	250	
Cr/Sc	120 nm Si_3_N_4_	3 × 5 mm								1.76	0.58	200	
Cr/Sc	120 nm Si_3_N_4_	3 × 5 mm	0.6 nm			18				2.04	0.49	200	
Ni/Ti	120 nm Si_3_N_4_	3 × 5 mm				6				2.33	0.33	100	
Mo/Si	890 nm SiN	5 × 5 mm		410 nm P–V (5 × 5 mm)		23	15	45	13.9			11	*n*
Mo/Si	100 nm SiN	5 × 5 mm	1.5–2 nm	4.4 nm r.m.s. / 38 nm P–V (3.2 mm diam)		23	7	7.2	13.9	7.3	0.4	20	*o*, *p*
Mo/Si	89 nm Si_3_N_4_	5 × 5 mm				14	15	45	14.68			9	*q*
Mo/Si	80 nm Si_3_N_4_	5 × 5 mm		25 nm r.m.s. (5 × 5 mm)	850	22			13.89			9	*r*
				4 nm r.m.s. (3 × 3 mm)									
Cr/C	Free-standing	10 × 10 mm	0.4 nm	160 nm P–V (8 × 8 mm)		3.3	5.6	45	6.36	4.34		50	*s*
						5.8	6.6	80	6.15	3.09		90	
Sc/Cr	Free-standing	8 mm diam	0.4 nm				0.4	59.77	3.11	3.05	0.47	300	*t*
Cr/C	Free-standing	10 mm diam					7	45	4.47	3.15	0.55	150	*u*
W/B_4_C	Free-standing	8 mm diam							1.46	1.38	0.44	350	*v*
W/B_4_C	Free-standing	8 mm diam				18	16	70	1.21	1.743	0.38	350	*w*
Zr/Si	Free-standing	20 × 140 mm		Not so flat			43		13	2.2	0.72	25	*x *
Zr/Si	Free-standing	33 × 38 mm					76		13	4.2	0.67	50	
Mo/C	Free-standing	33 × 38 mm					48		6.5	2.7	0.78	60	
Zr/Al	Free-standing	33 × 38 mm								8.7	0.59	11	
Cr/Sc	Free-standing	33 × 38 mm					39		2.5	3.1	0.48	63	
Al/Si	Free-standing	33 × 38 mm					39		17.1	4.7	0.68	65	
Mo/Si	50 nm SiN	3 × 3 / 10 × 10 mm	0.7–1 nm	4 nm r.m.s./32 nm P–V (3 × 3 mm) 22 nm r.m.s./200 nm P–V (10 × 10 mm)	∼100	Var.	Var.	Var.	13.5	Var.	0.63	5–30	FOM

† References: *a*: Stearns *et al.* (1986 ▶). *b*: Ceglio (1989 ▶). *c*: Khan Malek *et al.* (1989 ▶). *d*: Nomura *et al.* (1992 ▶). *e*: Kortright *et al.* (1992 ▶). *f*: Di Fonzo *et al.* (1994 ▶). *g*: Da Silva *et al.* (1995*a* ▶). *h*:Da Silva *et al.* (1995*b* ▶). *i*: Nguyen (1994 ▶). *j*: Tinone *et al.* (1996 ▶). *k*: Haga *et al.* (1998 ▶). *l*: Haga *et al.* (2000 ▶). *m*: Schäfers *et al.* (1999 ▶). *n*: Hubert *et al.* (2001 ▶). *o*: Wang *et al.* (2003 ▶). *p*: Wang *et al.* (2007 ▶). *q*: Smith *et al.* (2003 ▶). *r*: Zeitoun *et al.* (2004 ▶). *s*: Takenaka *et al.* (2005 ▶). *t*: Kimura *et al.* (2005 ▶). *u*: Andreev *et al.* (2005 ▶). *v*: MacDonald *et al.* (2008 ▶). *w*: MacDonald *et al.* (2009 ▶). *x*:Volodin *et al.* (2010 ▶).

**Table 2:** BS design criteria together with the main ML’s structure parameters for 13.5 nm wavelength applications

Experiment type	Incident angle	*R*, *T* criteria	Period (nm)	Number of bi-layers	Γ	Possible polarization
Pump–probe	15°	Maximum *R* *T*, *R* = 25%, *T* = 36%	7	12	0.5	s or p
Mach–Zehnder interferometer	47.5°	*R*:*T* ≃ 1:1, 29%	10.6	5	0.48	s
	17°	*R* = 35%, *T* = 25%	7.32	13	0.52	p
Maximum reflectivity over transmission ratio	12.5°	Maximum *R*, 55%	7.3	30	0.633	s or p
Fabry–Perot interferometer	12.5°	*R*:*T* ≃ 1:1, 29%	7.3	12	0.633	s
Fabry–Perot interferometer	12.5°	*R* = *T* ^2^ *R*_12_, *R* = 0.08, *T* = 0.52	7.3	5	0.633	s

**Table 3:** Mid-spatial-frequency surface roughness of a 10 × 10 mm uncoated membrane and coated with a different number of bilayers, with Γ = 0.63 and *d* = 7.3 nm

	r.m.s. / P–V (nm)	
*N* bilayers	20× magn	50× magn
0 (uncoated)	0.1 / 1	0.1 / 1
5	0.2 / 1.3	0.3 / 2.3
12	0.1 / 1.1	0.3 / 2.9
30	0.2 / 1.5	0.4 / 3.1

**Table 4:** The r.m.s. and P–V values of the profiles of uncoated membranes, as shown in Fig. 8 ▶, for the full frame, the whole window (10 × 10 mm) and zooms in the center of 5 and 1 mm

	r.m.s. / P–V (nm)		
	Uncoated 1	Uncoated 2	Uncoated 3
Full frame	139 / 934	324 / 1663	108 / 1390
Window (10 × 10)	43 / 391	59 / 436	25 / 246
Center (5 × 5)	9.6 / 63.6	6.5 / 41.4	4.5 / 33.5
Center (1 × 1)	0.7 / 3.6	0.6 / 3.6	0.6 / 3.2

**Table 5:** The r.m.s. and P–V values of the profiles of a BS in different holders, as shown in Fig. 9 ▶, for the full frame, the whole BS window (10 × 10 mm) and zooms in the center of 5 and 1 mm

	r.m.s. / P–V (nm)		
	Reference (loose support)	Clamps	Special holder
Full frame	112 / 672	1222 / 7422	157 / 1004
Window (10 × 10)	21.8 / 200	483 / 2914	35.1 / 312
Center (5 × 5)	3.2 / 21.4	123 / 740	6.4 / 38.1
Center (1 × 1)	0.88 / 4.6	5.7 / 30.4	0.67 / 3.43
